# Cardiac sodium channel regulator MOG1 regulates cardiac morphogenesis and rhythm

**DOI:** 10.1038/srep21538

**Published:** 2016-02-23

**Authors:** Juan Zhou, Longfei Wang, Mengxia Zuo, Xiaojing Wang, Abu Shufian Ishtiaq Ahmed, Qiuyun Chen, Qing K. Wang

**Affiliations:** 1 Key Laboratory of Molecular Biophysics of the Ministry of Education, Cardio-X Center, College of Life Science and Technology and Center for Human Genome Research, Huazhong University of Science and Technology, Wuhan, China; 2 Center for Cardiovascular Genetics, Department of Molecular Cardiology, Lerner Research Institute, Cleveland Clinic; Department of Molecular Medicine, Department of Genetics and Genome Sciences, Case Western Reserve University, Cleveland, Ohio, USA

## Abstract

MOG1 was initially identified as a protein that interacts with the small GTPase Ran involved in transport of macromolecules into and out of the nucleus. In addition, we have established that MOG1 interacts with the cardiac sodium channel Na_v_1.5 and regulates cell surface trafficking of Na_v_1.5. Here we used zebrafish as a model system to study the *in vivo* physiological role of MOG1. Knockdown of *mog1* expression in zebrafish embryos significantly decreased the heart rate (HR). Consistently, the HR increases in embryos with over-expression of human *MOG1*. Compared with wild type *MOG1* or control *EGFP*, mutant *MOG1* with mutation E83D associated with Brugada syndrome significantly decreases the HR. Interestingly, knockdown of *mog1* resulted in abnormal cardiac looping during embryogenesis. Mechanistically, knockdown of *mog1* decreases expression of *hcn4* involved in the regulation of the HR, and reduces expression of *nkx2.5*, *gata4* and *hand2* involved in cardiac morphogenesis. These data for the first time revealed a novel role that MOG1, a nucleocytoplasmic transport protein, plays in cardiac physiology and development.

*MOG1* was initially cloned from *Saccharomyces cerevisiae* while screening for a multi-copy suppressor of conditional growth defect alleles of the *Ran* gene (*GSP1*) (thus named as *mog1*)^1^. *Ran* encodes a small GTPase with a critical role in regulating the transport of macromolecules into and out of the nucleus through the nuclear pore complex (NPC)^2^. MOG1 directly interacts with Ran and can rescue the temperature sensitive growth defect phenotype associated with the yeast *Ran* mutant^3^. Similar to *Ran* mutant, yeast with ΔMOG1 was defective in nuclear-protein import^3^, suggesting that MOG1 is important for nucleocytoplasmic transport^3^. The human *MOG1* gene was identified by homology database searches. It codes for a protein of 186 amino acid residues with a predicted molecular weight of 20 kDa^4^. Human MOG1 protein sequence is highly homologous to that of yeast MOG1. Human MOG1 protein was shown to interact directly with Ran, too, and could partially rescue the growth defects of Δ*MOG1* yeast cells. The evolutionary conservation of the MOG1 protein sequence suggests that it also plays an important role in animal systems.

During a yeast two-hybrid screen, we identified MOG1 as a protein that also interacts directly with the voltage-gated cardiac sodium channel Na_v_1.5^5^. Na_v_1.5, encoded by the *SCN5A* gene, is required for generation and maintenance of the cardiac action potential (CAP)^6^,^7^,^8^,^9^,^10^,^11^. We have identified the first series of mutations in *SCN5A* which cause inherited cardiac arrhythmias and sudden death in the young, otherwise healthy, individuals, including long QT syndrome (LQTS) and Brugada syndrome (BrS)^6^,^12^,^13^. Later, *SCN5A*/Na_v_1.5 mutations were also found in patients with sick sinus syndrome, cardiac conduction disease, dilated cardiomyopathy and other diseases (together referred to as sodium channelopathies)^14^,^15^,^16^. We have reported that overexpression of *MOG1* promotes cell surface trafficking of Na_v_1.5, thereby increasing the density of peak cardiac sodium current (*I*_*Na*_), whereas siRNA knockdown of *MOG1* expression reduces cell surface trafficking of Na_v_1.5, thereby decreasing the density of *I*_*Na*_^5^,^17^. One dominant-negative mutation, E83D, of MOG1 was found to be associated with BrS and dramatically decrease *I*_*Na*_^18^. We recently found that overexpression of *MOG1* can rescue biochemical and electrophysiological defects associated *SCN5A*/Na_v_1.5 mutations causing BrS and sick sinus syndrome in mammalian cells^17^. Therefore, *MOG1*, delivered by gene therapy, may be used as an effective tool to treat patients with BrS and sick sinus syndrome with *SCN5A*/Na_v_1.5 mutations and other cardiac diseases with reduced *I*_*Na*_.

Despite the important roles of MOG1 in human diseases and potential utility in treating lethal arrhythmias, little is known about the *in vivo* function of *MOG1*. To address this issue, we used zebrafish as a model system to examine the role of *MOG1* in the developing heart. Zebrafish embryos are optically transparent and externally fertilized, making them ideal for studying early organogenesis. Moreover, genetic manipulation is easily achieved using antisense morpholinos (MOs) and embryos are permeable to small molecule drugs placed in the medium.

The stages of zebrafish cardiac development have been well-delineated. Cardiac precursors are located at the blastula margin, and development of the zebrafish heart begins at 5 hours post fertilization (hpf)^19^. These bilateral precursors undergo a complex series of movements that result in the formation of a cardiac cone and subsequently a beating heart tube by 22–24 hpf 20,21. The tube then loops to form a two-chambered heart and the ventricular wall begins to thicken concentrically between 48–72 hpf 20,21. In this study, we identified an important role of *mog1* in early cardiac development by regulating the expression of early transcription factor genes *nkx2.5*, *gata4*, and *hand2*. Moreover, we found that Mog1 regulates the heart rate during embryogenesis by regulating the expression of ion channel gene *hcn4*.

## Results

### Molecular cloning and expression profile of zebrafish *mog1*

**Figure 1:**
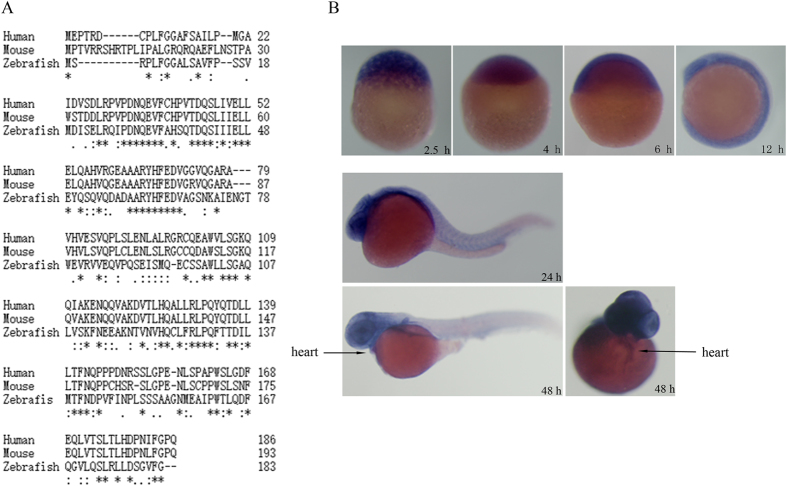
Identification of zebrafish *mog1* gene and its expression profile. (**A**) Alignment of amino acid sequences of human MOG1 (GenBankTM accession number NP_057576), mouse Mog1 (GenBankTM accession number NP_001272370) and zebrafish Mog1 (GenBankTM accession number NP_001093465). (**B**) Whole-mount *in situ* hybridization analysis of zebrafish *mog1* expression at different developmental stages during embryogenesis. Images for embryos at 2.5 hpf, 4 hpf, 6 hpf and 12 hpf are shown with lateral views with the animal pole to the top. Images for embryos at 24 hpf and 48 hpf stages are lateral views with the head to the left. A dorsal view of the 48 hpf embryo was also shown with the head to the top.

Using the human *MOG1* cDNA sequence, we performed BLAST searches for its homologous genes in the NCBI database (www.ncbi.nlm.nih.gov) and identified a single zebrafish *mog1* gene (RefSeqDNA: NM_001099995.1). Its corresponding protein sequence (RefSeq peptide: NP_001093465) showed a high degree of homology to human MOG1 (NP_057576) (46% identity, 62% homology) and mouse Mog1 (NP_001272370) (44% identity, 57% homology) (Fig. 1A and Table 1). Zebrafish *mog1* is located on chromosome 5 and contains 5 exons and 4 introns. The zebrafish Mog1 protein contains 183 amino acids.

The expression profile of *mog1* during zebrafish embryogenesis was analyzed using whole-mount *in situ* hybridization (Fig. 1B). Expression of *mog1* was detected as early as in one-cell-stage embryos, suggesting that *mog1* is maternally expressed. From 2.5 hpf to 4 hpf, *mog1* RNA was localized in blastomeres. From 6 hpf to 12 hpf, *mog1* expression was ubiquitous in embryos. At 24 hpf, *mog1* expression was over the entire embryo, but more prominently in the brain region and eyes. At 48 hpf*, mog1* was expressed predominantly in the brain area, but its expression was also detected in the heart and fins (Fig. 1B).

### Knockdown of *mog1* expression decreases the heart rate (HR)

**Figure 2:**
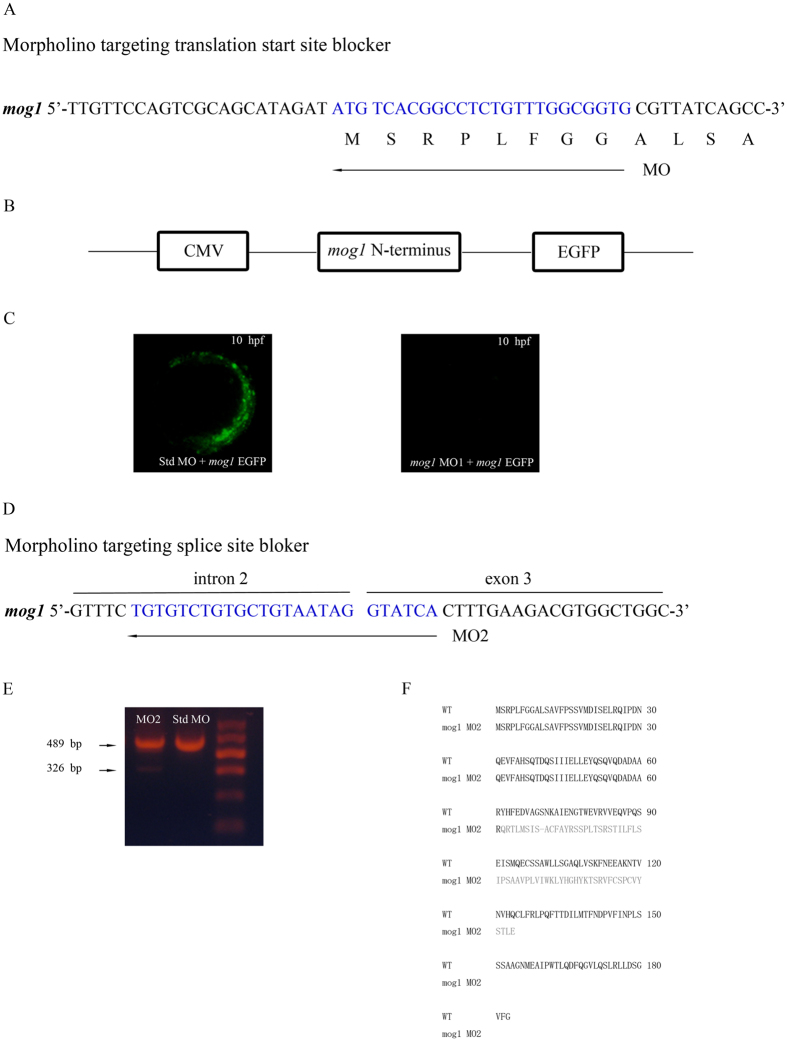
Two *mog1* MOs effectively knock expression of *mog1* down. (**A**) *Mog1* translation-blocking MO1 was directed against the translation start site. (**B**) Construction of a pCMV-mog1-EGFP reporter. The 5′-UTR of zebrafish *mog1* and a part of the *mog1* coding sequence (nucleotide sequences 1–171 starting with A of ATG start codon, amino acid residues 1–57) were fused to the *EGFP* gene in vector pEGFP-N1, in which expression of the Mog1–EGFP fusion protein (green) is under the control of the *CMV* promoter. (**C**) The *mog1*-EGFP reporter gene was injected into one- to two-cell-stage embryos together with control Std-MO or *mog1* MO1. Expression of the Mog1–EGFP fusion protein was examined under a fluorescence microscope at the 10 hpf stage. Note that *mog1* MO1 effectively abolished the expression of MOG1–EGFP (lack of green signal) compared with control. (**D**) *Mog1* splicing-blocking MO2 was designed to target the intron 2–exon 3 splicing donor site. (**E**) Results of RT–PCR analysis with total RNA from 24 hpf embryos treated with MO2 or Std MO. MO2 embryos generated a smaller size of alternatively spliced RT–PCR product (326 bp in MO2 versus 489 bp in Std MO). (**F**) Sequence analysis of the 326 bp band from MO2-injected embryos revealed that MO2 resulted in an alternatively spliced *mog1* transcript with a deletion of exon3 (163 bp).

To investigate the *in vivo* role of *mog1*, we knocked the expression of *mog1* down in zebrafish using two independent morpholinos (MOs), one MO targeting the translation start site (MO1) (Fig. 2A) and the other targeting a splicing site spanning intron 2 and exon 3 (MO2) (Fig. 2D). Utilization of two independent MOs may avoid a potential off-target effect of MOs. To examine the effectiveness of MO1, we cloned a *mog1* cDNA fragment containing the 5′-UTR, the translation start codon ATG, and the N-terminal coding region (amino acid residues 1 to 57) in the front of *EGFP* and under the control of the *CMV* promoter, resulting in a *mog1*–*EGFP* reporter (Fig. 2B). The effectiveness of MO1 was examined by injecting MO1 together with the *mog1*–*EGFP* reporter plasmid DNA into 1–2 cell embryos. The EGFP signal (*mog1* expression) in the embryos injected with MO1 was markedly decreased by 95% (n = 60) compared to that in the embryos injected with control Std MO (n = 69) (Fig. 2C). These data suggest that MO1 is highly effective in knocking *mog1* expression down.

To test the effectiveness of MO2, we injected MO2 or Std MO into 1–2 cell embryos. RT-PCR analysis of mRNAs from the embryos followed by Sanger sequencing analysis revealed that MO2 injection led to an alternatively spliced transcript without exon 3, resulting in a frameshift mutation and premature translation stop (Fig. 2D–F). The amount of the alternatively spliced transcript (lower 326 bp band) generated by MO2 was considerably less than that of the wild type full-length transcript (upper 489 bp band), but it is clearly visible and consistently identified in every experiment (n > 3) (Fig. 2E). These data suggest that MO2 can knock *mog1* expression down.

**Figure 3:**
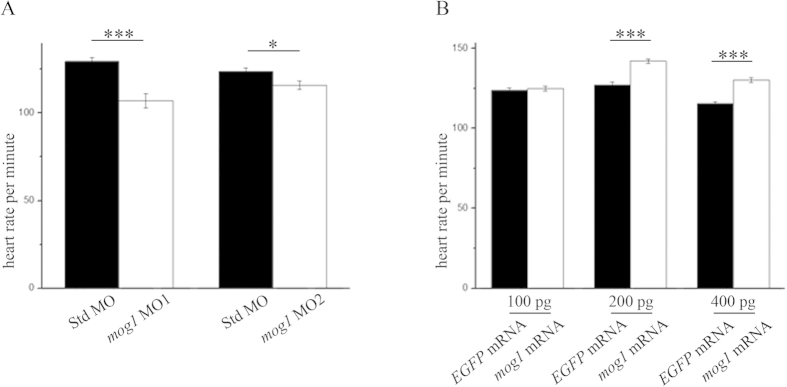
Mog1 regulates the heart rate (HR). (**A**) Knockdown of *mog1* expression by *mog1* MO1 and MO2 caused reduction of the HR at 72 hpf. HR: 107 ± 4 bpm, n = 31 for *mog1* MO1 morphants; 129 ± 4 bpm, n = 35 for control Std MO morphants (***P < 0.0001); 115 ± 2 bpm, n = 22 for *mog1* MO2 zebrafish; 123 ± 2 bpm, n = 21 for control Std MO zebrafish (*P < 0.05). (**B**) Overexpression of *mog1* in zebrafish embryos caused an increase of the HR at 120 hpf. HR at a dose of 100 pg: 124 ± 1 bpm, n = 33 embryos for *mog1* mRNA; 123 ± 1 bpm, n = 35 embryos for control *EGFP* mRNA (*P* > 0.05). HR at a dose of 200 pg: 142 ± 1 bpm, n = 44 embryos for *mog1* mRNA; 127 ± 2bpm, n = 33 embryos for control *EGFP* mRNA (***P < 0.0001). HR at a dose of 400 pg: 130 ± 2 bpm, n = 39 embryos for *mog1* mRNA, 115 ± 1 bpm, n = 37 embryos for control *EGFP* mRNA (***P < 0.0001). Data were shown as means ± SEM from three independent experiments. We have noted that the heart rate varies from one experiment to another experiment (probably due to fluctuation of temperature), therefore, the comparison of the heart rate was always made between two groups in the same set of experiments.

In control embryos injected with Std MO, the heart exhibited vigorous, rhythmic contractions. However, *mog1* morphants with injection of *mog1* MO1 showed bradycardia, and the myocardial contraction was obviously weakened. The HR was manually counted. Knockdown of *mog1* expression by MO1 significantly decreased the HR compared to control embryos. At 72 hpf, the HR was 18% lower in *mog1* MO1 zebrafish (106 ± 4 bpm, n = 31 embryos) than that in control zebrafish (129 ± 2 beats per minute or bpm, n = 35 embryos) (*P* = 2.27×10^−5^) (Fig. 3A). Similar but less dramatic results were obtained with *mog1* MO2 (115 ± 2 bpm, n = 22 embryos vs. control MO (123 ± 2 bpm, n = 21 embryos) (*P* = 0.02) (Fig. 3A).

### Overexpression of zebrafish *mog1* gene increases the HR

To further confirm the finding that *mog1* regulates the HR, we overexpressed *mog1* in zebrafish embryos by direct injection of *mog1* mRNA (200 pg). At 72 hpf, no statistical difference was found on the HR between embryos with injection of *mog1* mRNA and those with control *EGFP* mRNA. However, at 120 hpf, the HR of embryos injected with *mog1* mRNA (142 ± 1 bpm, n = 44 embryos) was significantly increased compared to that of control embryos injected with *EGFP* mRNA (127 ± 2 bpm, n = 33 embryos) (*P* = 1.12 ×10^−8^) (Fig. 3B). Similar results were obtained at a higher dose of *mog1* mRNA (400 pg) (*P* = 1.02 ×10^−10^) (Fig. 3B).

### Mog1 regulates expression of *hcn4* during embryogenesis in zebrafish

**Figure 4:**
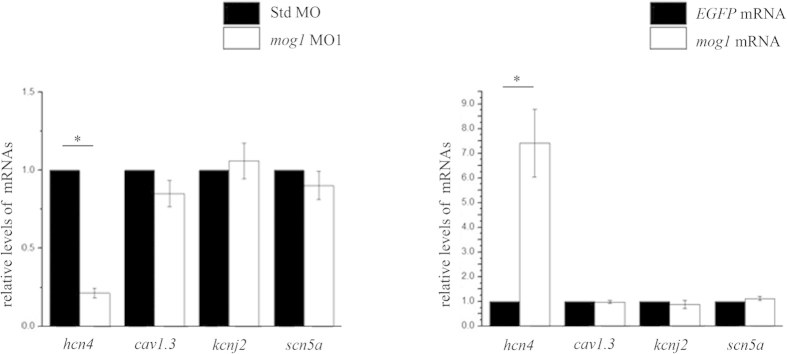
Mog1 regulates expression of *hcn4*. (**A**) Real-time quantitative RT-PCR analysis of *hcn4*, *cav1.*3, *kcnj2*, and *scn5a* in *mog1* MO1 morphants or control Std MO morphants. (**B**) Real-time quantitative RT-PCR analysis of *hcn4*, *cav1.*3, *kcnj2*, and *scn5a* in zebrafish embryos injected with *mog1* mRNA or control *EGFP* mRNA. Data were shown as means ± SEM from three independent experiments (each in triplicate).

The heart rhythm is generated and maintained by various ionic currents^22^,^23^, including *I*_*f*_ generated by HCN4^24^,^25^,^26^,^27^,^28^ and the cardiac calcium current generated by CAV1.3^29^,^30^. Studies with transgenic overexpression mice and genetic studies of human patients with sick sinus syndrome suggested that *I*_*K1*_ generated by KCNJ2^31^ and the cardiac sodium current generated by Na_v_1.5 (encoded by *SCN5A*)^32^,^33^ were also involved in regulation of the HR, respectively. To explore the molecular mechanism by which Mog1 regulates the HR, we performed real time qRT-PCR analysis for *hcn4, cav1.3, kcnj2* and s*cn5a* in zebrafish embryos injected with MO1. Total RNA was isolated from zebrafish embryos injected with *mog1* MO1 or control Std MO and used for real-time qRT-PCR analysis. As shown in Fig. 4A, injection of *mog1* MO1 selectively reduced the expression level of *hcn4* mRNA in zebrafish embryos, but not that of *kcnj2*, *cav1.3*, and *scn5a* (Fig. 4A). Consistently, overexpression of *mog1* by injection of *mog1* mRNA markedly increased the expression of *hcn4*, but did not have any effect on expression of *kcnj2*, *cav1.3* or *scn5a* (Fig. 4B). Together, these data suggest that Mog1 is involved in regulating expression of *hcn4*.

### Knockdown of *mog1* expression results in defects in cardiac morphogenesis

**Figure 5:**
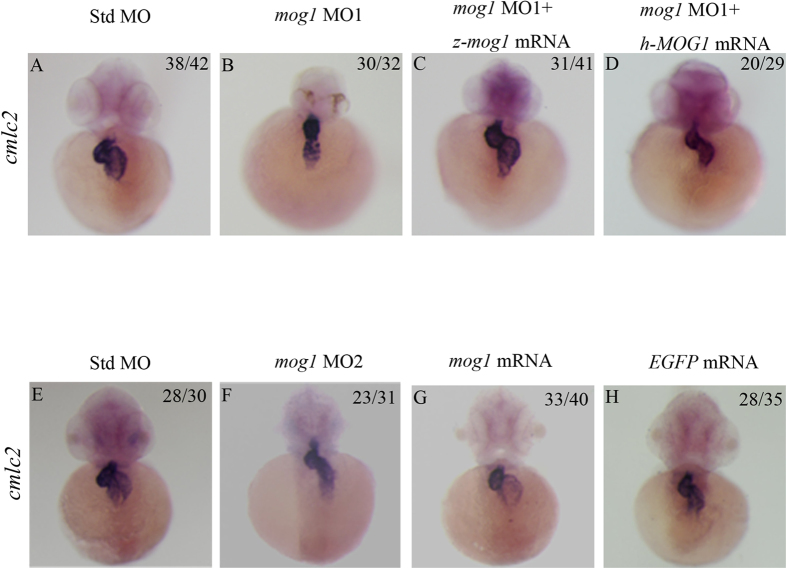
Mog1 regulates cardiac morphogenesis and development during zebrafish embryogenesis. (**A,B**) Whole-mount *in situ* hybridization with embryos injected with 16 ng of control Std-MO (**A**) or 16 ng of *mog1* MO1 (**B**) with a cardiac marker, *cmlc2*, at 48 hpf. (**C,D**) Whole-mount *in situ* hybridization with embryos injected with 16 ng of *mog1* MO1 together with 200 pg of zebrafish *mog1* mRNA (**C**) or with 200 pg of human *MOG1* mRNA (**D**) with a cardiac marker, *cmlc2*, at 48 hpf. Note that overexpression of *mog1* rescued the defects in *mog1* morphants. The construct for making zebrafish *mog1* mRNA was mutated at 5 positions (from ATG TCA CGG CCT CTG TTT to ATG TCT CGT CCG CTA TTC) so that *mog1* MO1 can knock endogenous zebrafish *mog1* mRNA down, but not the *in vitro* synthesized *mog1* mRNA used for injection. The *mog1* MO1 cannot bind to human MOG1 mRNA so that it cannot knock the *in vitro* synthesized human *MOG1* mRNA down. (**E,F**) Whole-mount *in situ* hybridization with embryos injected with 16 ng of control Std-MO (**E**) or 16 ng of *mog1* MO2 (**F**) with a cardiac marker, *cmlc2*, at 48 hpf. (**G,H**) Overexpression of *mog1* did not cause changes of cardiac phenotype compared with embryos injected with *EGFP* mRNA (negative controls). The numbers in the upper right corner (e.g. 38/42 in **A**) describe the ratio of the number of embryos with the phenotype (e.g. 38) in the image over the number of total embryos analyzed (e.g. 42). Scale bar = 100 μM.

To determine if knockdown of *mog1* affects the structure and/or morphology of the heart, we performed whole-mount *in situ* hybridization for *cmlc2*, a marker for cardiomyocytes. *Mog1* MOs caused abnormal cardiac looping, a key step in development of the heart (Fig. 5). Co-injection of *mog1* MO1 (16 ng) and *in vitro* synthesized, capped zebrafish *mog1* mRNA or human *MOG1* mRNA (200 pg) into 1–2 cell embryos rescued the defects of cardiac looping caused by *mog1* MO1 at 48 hpf (compare Fig. 5A–D). Similar results were obtained with *mog1* MO2 (Fig. 5E,F), although the effect of MO2 was less severe than that of MO1. These results suggest that *mog1* plays an important role in heart development during embryogenesis.

Overexpression of *mog1* by injection of *mog1* mRNA into zebrafish embryos did not cause apparent changes in cardiac morphogenesis and development (Fig. 5G,H). This may be due to the finding that the endogenous *mog1* level was found to be high^4^ so that further overexpression did not have any effect on cardiac morphogenesis and development.

### Knockdown of *mog1* reduces expression of cardiac transcription factors in the anterior lateral mesoderm

**Figure 6:**
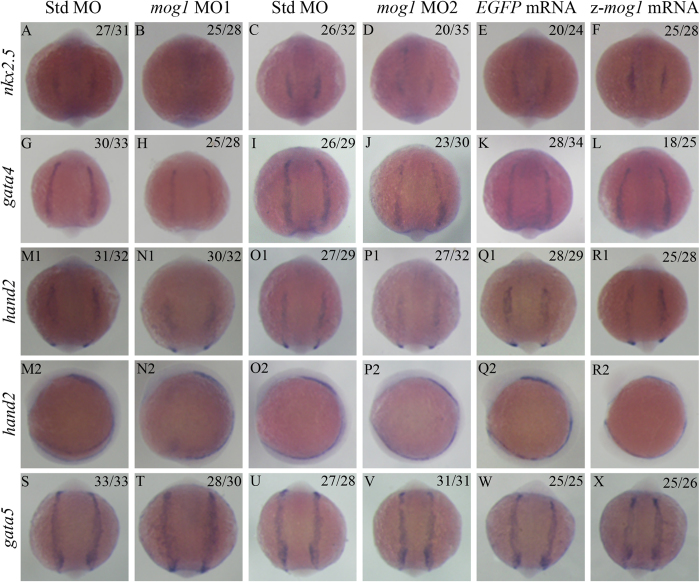
Knockdown of *mog1* causes a decrease of expression levels of cardiac transcriptional factors in the anterior lateral mesoderm by whole-mount *in situ* hybridization. (**A–D**) Knockdown of *mog1* expression reduces intensity of the *nkx2.5* signal. Zebrafish embryos (1–2 cell stage) were injected with 16 ng of Std-MO (**A,C**), 16 ng of *mog1* MO1 (**B**) or 16 ng of *mog1* MO2 (**D**) and used for whole-mount *in situ* hybridization at 12 hpf with an antisense probe for *nkx2.5*. (**G–J**) Knockdown of *mog1* expression reduces intensity of the *gata4* signal. The embryos were injected and processed as in (**A–D**), but probed with an antisense probe for *gata4*. (**M**–**P**) Knockdown of *mog1* expression reduces expression of *hand2*. (**S**–**V**) Knockdown of *mog1* expression does not affect expression of *gata5*. (**E**,**F**,**K**,**L**,**Q**,**R**,**W**,**X**) Over-expression of zebrafish *mog1* did not affect expression of cardiac transcriptional factors in the anterior lateral mesoderm at the 12 hpf stage. (**A**–**R1**,**S–X**) Embryos are shown at a dorsal view with the anterior at the top. (**M2**–**R2**) embryos are shown in a lateral view with the anterior on the left. The numbers in the upper right corner (e.g. 27/31 in **A**) describe the ratio of the number of embryos with the phenotype (e.g. 27) in the image over the number of total embryos analyzed (e.g. 31). Scale bar = 100 μM.

**Figure 7:**
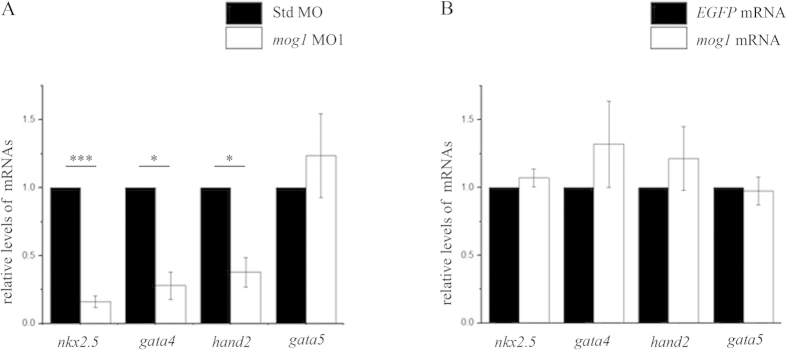
Knockdown of *mog1* causes a decrease of expression levels of cardiac transcriptional factors in the anterior lateral mesoderm by real-time quantitative RT-PCR analysis. (**A**) Knockdown of *mog1* expression significantly reduced the expression level of *nkx2.5* (***P < 0.0001), *gata4* (*P < 0.05) and *hand2* (*P < 0.05), but not that of *gata5* (*P* > 0.05). (**B**) Overexpression of zebrafish *mog1* by injection of 200 pg mRNA did not affect the expression level of *nkx2.5* (*P* > 0.05), *gata4* (*P* > 0.05), *hand2* (*P* > 0.05) or *gata5* (*P* > 0.05). Std MO (**A**) was used as control for MO1 and *EGFP* mRNA (**B**) was used as control for *mog1* mRNA. Data were shown as means ± SEM from three independent experiments (each in triplicate).

To identify the molecular mechanism by which *mog1* regulates cardiac morphogenesis and development, we performed whole-mount *in situ* hybridization to examine expression of important cardiac transcription factors in the heart-forming region of the anterior lateral plate mesoderm (ALPM). The ALPM is a population of undifferentiated cells with a cardiac potential demarcated by the expression of *nkx2.5*^34^. The *gata4* and *hand2* genes are also expressed in the ALPM, but their expression regions are larger than that for *nkx2.5*^34^. Compared to control embryos, *mog1* morphants with injection of MO1 displayed a decreased expression level of all three transcription factors in the ALPM at the 6-somite stage (Fig. 6A–D,G–J,M–Q). Somite staging was used to confirm that the observed differences were not the result of developmental delay. Although *gata5* is a potent inducer of *nkx2.5* expression in zebrafish^35^, its expression level was indistinguishable between *mog1* morphants and control embryos (Fig. 6S–V). These results were independently corroborated by real time qPCR analysis. Knockdown of *mog1* led to a significant decrease in the expression levels of *nkx2.5*, *gata4*, and *hand2* (Fig. 7A). These results indicate that zebrafish *mog1* is required for the normal expression of cardiac fate-determining genes in the ALPM and for specification of appropriate numbers of cardiac progenitor cells during development.

Over-expression of zebrafish *mog1* with injection of *mog1* mRNA, however, did not cause any changes in expression of *nkx2.5, gata4, hand2*, and *gata5* in the ALPM (Fig. 6E,F,K,L,Q,R,W,X). Real time qPCR analysis did not identify any effect of *mog1* overexpression on expression of *nkx2.5, gata4, hand2*, and *gata5*, either (Fig. 7B).

**Figure 8:**
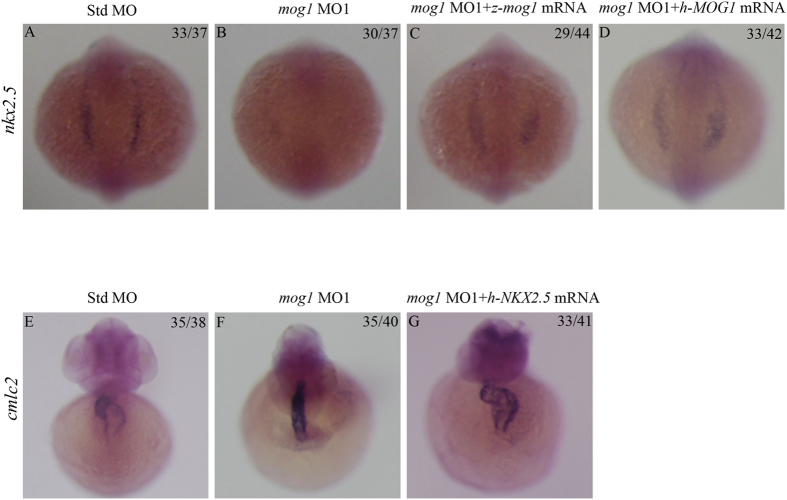
Cardiac developmental defects in *mog1* morphants are rescued by overexpression of *nkx2.5*. Zebrafish embryos were injected with Std-MO (16 ng; **A**,**E**), *mog1* MO1 (16 ng; **B**,**F**), *mog1* MO1 (16 ng) together with *in vitro* synthesized full-length zebrafish (**C**) or human *MOG1* mRNA (200 pg) (**D,G**) and used for whole-mount *in situ* hybridization at 12 somites with antisense probes for *nkx2.5* (**A–D**) or *cmlc2* (**E–G**). Reduced expression of *nkx2.5* by knockdown of *mog1* was rescued by injection of *mog1* mRNA (**A–D**). Co-injection of *nkx2.*5 mRNA (**G**) rescued the cardiac developmental defects in *mog1* MO1 morphants (whole-mount *in situ* hybridization signal for *cmlc2*). (**A–D**) Dorsal view with the anterior at the top. (**E–G**) Anterior view with the ventral side at the top. The numbers in the upper right corner (e.g. 33/37 in **A**) describe the ratio of the number of embryos with the phenotype (e.g. 33) in the image over the number of total embryos analyzed (e.g. 37).

To determine if abnormal cardiac looping in embryos injected with the *mog1* MO was caused by decreased expression of cardiac transcriptional factors, we co-injected zebrafish *nkx2.5* mRNA (80 pg) together with *mog1* MO1 into 1–2 cells stage embryos. At 48 hpf, co-injection of human *nkx2.5* mRNA rescued the defects of cardiac morphogenesis caused by *mog1* MO1 (Fig. 8E–G). Furthermore, the reduced expression level of *nkx2.5* at the 6 somite stage caused by *mog1* MO1 was partially rescued by both human *MOG1* or zebrafish *mog1* mRNA injection (Fig. 8A–D). These results suggest that *mog1* acts upstream of *nkx2.5* to regulate specification of cardiac morphogenesis.

### Brugada syndrome mutation E83D in *mog1* reduces the heart rate

**Figure 9:**
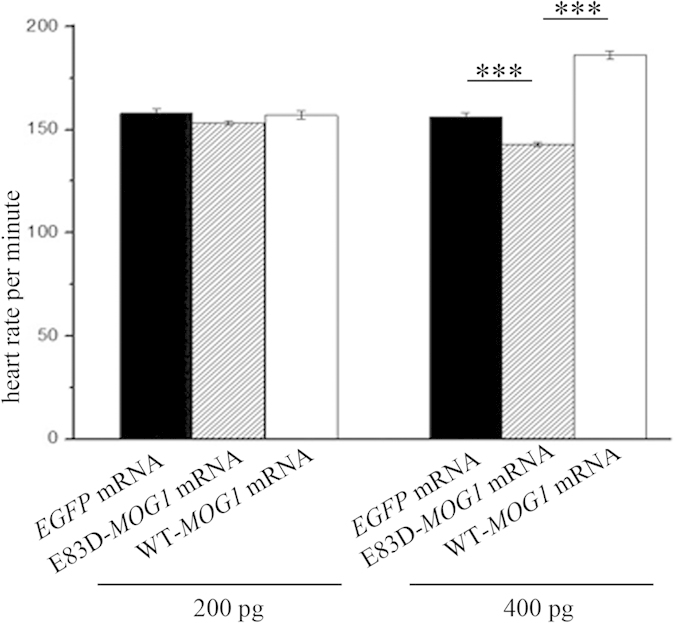
Brugada syndrome mutation E83D in *MOG1* reduces the HR. (**A**) HR at the dose of 200 pg mRNA (158 ± 2 bpm, n = 29 for EGFP embryos vs. 153 ± 1 bpm, n = 36 for E83D embryos vs. 157 ± 2 bpm, n = 34 for WT embryos, *P* > 0.05 at day 5). (**B**) HR at the dose of 400 pg mRNA (156 ± 2 bpm, n = 32 for EGFP embryos vs. 143 ± 1 bpm, n = 30 for E83D embryos, ***P < 0.0001; 143 ± 1 bpm, n = 30 for E83D embryos vs. 186 ± 2 bpm, n = 29 for WT embryos ***P < 0.0001). Data were shown as means ± SEM from three independent experiments.

Previously, a mutation in human *MOG1* was identified in a symptomatic female patient with BrS, a cardiac disorder characterized by ST segment elevation in right precordial leads on electrocardiograms (ECGs), syncope, ventricular arrhythmia, and sudden cardiac death (SCD)^6^,^18^. Here we tested whether MOG1 mutation E83D affected the HR in zebrafish embryos. We injected mRNA samples transcribed from a wild type human *MOG1* expression plasmid or from a mutant human *MOG1* expression plasmid with the E83D mutation into 1–2 cells stage embryos, respectively. At the dose of 200 pg mRNA, no statistical difference was found on the HR between the embryos over-expressing the E83D mutation and those over-expressing wild type MOG1 or with injection of control *EGFP* mRNA (153 ± 1 bpm, n = 36 E83D embryos vs. 157 ± 2 bpm, n = 34 WT embryos or 158 ± 2, n = 29 EGFP embryos, *P* = 0.275) at day 5 (Fig. 9A). However, at the dose of 400 pg mRNA, embryos with overexpression of the E83D mutant MOG1 showed a significantly reduced HR compared to that of the embryos with overexpression of wild type MOG1 (143 ± 1 bpm, n = 30 E83D embryos vs. 186 ± 2 bpm, n = 29 embryos *P* = 1.27×10^−36^) or to embryos with injection of control *EGFP* mRNA (143 ± 1 bpm, n = 30 E83D embryos vs. 156 ± 2 bpm, n = 32 embryos, *P* = 2.09×10^−9^) (Fig. 9B). Thus, mutant MOG1 with the BrS-associated E83D mutation caused a decrease of the HR in zebrafish.

## Discussion

Using zebrafish as an *in vivo* system, we have identified a novel role for Mog1 in cardiac rhythm and morphogenesis. Knockdown of *mog1* by two independent morpholinos significantly decreased the HR, whereas overexpression of *mog1* by direct injection of *mog1* mRNA into the embryos increased the HR. We then showed that knockdown of *mog1* expression in zebrafish caused a decrease of *hcn4* expression without affecting expression of other ion channel genes, including *kcnj2, cav1.3 or scn5a*. Furthermore, overexpression of *mog1* in zebrafish led to an increased level of *hcn4* expression. Hcn4 is the dominant Hcn isoform in the adult sinoatrial node of all species investigated (rabbit, mouse, dog)^25^. This channel mediates the sympathetic stimulation of the HR in embryos. It contributes significantly to the generation of the regular pacemaker potential and the determination of the basal HR^24^,^26^,^27^. Mutations in *HCN4* cause sick sinus syndrome in humans^27^,^28^. Loss of *Hcn4* led to severe bradycardia and chronotropic incompetence in the embryos of knockout mice^26^. Results of these studies are in line with our observations and suggest that Mog1 regulates the cardiac rhythm in zebrafish through regulation of *hcn4* expression.

However, the molecular mechanism underlying the regulation of *hcn4* expression by *mog1* is unknown. *Mog1* is involved in regulating nucleocytoplasmic transport of proteins, and it may regulate nuclear import of transcriptional factors and RNA splicing factors and nuclear export of regulators of translation, some of which may be directly involved in the regulation of *hcn4* transcription, splicing or translation. Future studies should focus on detailed investigations of these interesting possibilities.

Unexpectedly, we found that knockdown of *mog1* by MOs significantly altered cardiac morphogenesis and development, resulting in abnormal hearts failing to complete looping. In zebrafish, both *gata4* and *hand2* are expressed in larger domains of the ALPM than *nkx2.5*^34^,^36^,^37^. Prior studies in zebrafish revealed essential roles for Nkx2.5 in limiting the atrial cell number, promoting the ventricular cell number, and preserving chamber-specific identity in differentiated myocardium^38^. Nkx2.5 is essential during cardiac progenitor differentiation to maintain ventricular and atrial chamber morphology and cellular traits later in development^39^. Reduced *gata4* expression was observed to cause defects in cardiac chamber growth and looping, while loss of *hand*2 resulted in embryos with significantly fewer embryonic cardiomyocytes^36^,^37^. We have demonstrated that knockdown of *mog1* expression led to a significant decrease of the expression levels of multiple cardiac transcription factor genes, including *nkx2.5*, *gata4*, and *hand2* in the ALPM (Fig. 6). Consistently, the expression of *mog1* was identified in the gastrulating embryo, prior to the differentiation of excitable tissues (Fig. 1). By 12 hpf, the cardiac-specification gene *Nkx2.5* identifies bilateral stripes of cardiac progenitors in the ALPM. Zebrafish in which *mog1* was knocked down exhibited defects in initial specification of the pre-cardiac mesoderm as shown by marked reduction in early expression of Nkx2.5. Previous studies in zebrafish have demonstrated that *gata5* is a potent positive regulator of *nkx2.5* expression^35^,^40^. Notably, we found that although *mog1* regulated *nkx2.5* expression, it did not affect the expression level of *gata5*. On the other hand, we found that *mog1* regulated expression of *gata4*, which may be a key to regulation of *nkx2.5*. All together, these data suggest that *mog1* may regulate cardiac morphogenesis and development by modulating the expression levels of *nkx2.5*, *gata4*, and/or *hand2*.

The abnormal cardiac looping detected in *mog1* morphants was remarkably similar to that observed in embryos with knockdown of *scn5a* expression by MOs reported previously^32^. When the findings in *scn5a* morphants were reported, it was puzzling why down-regulation of an ion channel gene *scn5a* caused abnormalities in cardiac morphogenesis and development. The puzzle may be partially resolved with our findings in this study that knockdown of *mog1* expression also caused abnormal cardiac morphogenesis and development. We have identified MOG1 as an interacting protein of Na_v_1.5 by a yeast two hybrid screen^5^. MOG1 regulates the function of Na_v_1.5. Overexpression of *MOG1* increases peak cardiac sodium current density, whereas knockdown of *MOG1* decreased the density of peak sodium current^5^,^17^. Mechanistically, MOG1 facilitates trafficking of Na_v_1.5 to plasma membrane and caveolae on the cell surface^17^. Conversely, it is likely that knockdown of *scn5a* expression by MOs may affect the function of Mog1 because both of them interact strongly and may cross-regulate their functions. Deregulation of functions of *mog1* by *scn5a* MO may affect expression of cardiac transcriptional factors *nkx2.5*, *gata4* and *hand2*, resulting in abnormal cardiac morphogenesis and development.

Kattygnarath *et al.* identified a missense mutation, E83D, in *MOG1* in a BrS patient^18^. Wild type MOG1 significantly increased the peak sodium current density (−171.0 ± 19.0 pA/pF at −20 mV for WT-MOG1 vs. −91.5 ± 11.6 pA/pF at −20 mV for empty vector)^18^. The E83D mutation significantly reduced the sodium current density (−89.5 ± 28.4 pA/pF) compared with wild type MOG1, and co-expression of both wild type and mutant MOG1 failed to increase sodium density (−106.7 ± 6.1 pA/pF)^18^. Despite the strong functional data, Olesen *et al.*^41^ raised a cautious note about the causal nature of the mutation due to identification of the mutation in a single BrS patient and lack of co-segregation analysis. In this study, we found that injection of wild type human *MOG1* mRNA increased the HR in zebrafish embryos, but injection of mutant *MOG1* mRNA with the E83D mutation resulted in a significantly lower HR than wild type *MOG1* mRNA. Our data provides further evidence that the E83D mutation in MOG1 is a functional mutation affecting MOG1 function.

There are several limitations with the current study. First, although two independent *mog1* MOs were studied to minimize the confounding of off-target effects of MOs, the second MO, i.e. MO2 targeting a splicing junction, was considerably less effective than MO1 targeting the translation start site. Nevertheless, similar results were obtained for both MO1 and MO2. Second, our studies could not distinguish whether the two major effects of *mog1* MOs, i.e. abnormal cardiac looping and a decreased HR, are two independent events or related. Because *mog1* MOs reduced the expression levels of *hcn4* and transcriptional factor genes *nkx2.5*, *gata4*, and *hand2*, respectively, it is likely that abnormal cardiac looping and a decreased HR caused by MOs may be two independent events. However, we could not exclude the possibility that abnormal cardiac looping may precipitate a decreased HR because knockdown of *mog1* expression reduced the expression levels of *nkx2.5*, *gata4*, and/or *hand2* (causing abnormal cardiac looping), which may decrease expression of hcn4 (reducing the HR).

In conclusion, the data in this study demonstrates that Mog1 regulates heart rhythm through regulating the expression of potassium channel Hcn4. In addition, Mog1 also regulates cardiac morphogenesis and development through regulating expression of cardiac transcriptional factors Nkx2.5, Gata4, and Hand2. We also showed that BrS-associated mutation E83D in MOG1 significantly reduced the HR compared with wild type MOG1, providing *in vivo* evidence that E83D is a functional mutation. These studies uncover a novel physiological role of Mog1 in regulating heart rhythm and morphogenesis *in vivo* and further our understanding of molecular mechanisms and biological pathways for maintenance of appropriate heart rhythm and for regulation of cardiac morphogenesis and development.

## Methods

### Zebrafish breeding and maintenance

Wild type AB strain zebrafish *(Danio rerio)* were used in the study. Embryos were raised at 28.5 °C, treated with 0.003% 1-phenyl-2-thiourea (PTU, Sigma) to prevent pigment formation, and collected at different developmental stages for analysis^42^,^43^. The study was approved by the ethics committee of Huazhong University of Science and Technology, and all procedures were carried out in accordance with the approved guidelines.

### Identification and homology analysis of zebrafish *mog1*

The full-length sequence of zebrafish *mog1* gene was identified by searching the NCBI database (http://www.ncbi.nlm.nih.gov). BLAST searches were performed to identify potential homologs, othologs or paralogs of zebrafish *mog1*. In zebrafish, there is only one copy of the *mog1* gene and no paralog was found.

### Microinjection of morpholinos (MOs)

To investigate the *in vivo* role of *mog1*, we studied two different *mog1* MOs to knock expression of *mog1* down in zebrafish embryos. The first MO targets the translation start site of *mog1* (MO1: 5′- CACCGCCAAACAGAGGCCGTGACAT -3′) (Fig. 1A), and the second MO targets the splicing junction between the second intron and the third exon (MO2: 5-TGATACCTATTACAGCACAGACACA -3′) (Fig. 1B). The negative control MO is a standard control (Std) morpholino that does not match any zebrafish sequence (5′-CCTCTTACCTCAGTTACAATTTATA-3′). MOs (16 ng) were dissolved in water and injected into the yolk sac of one- to two-cell stage zebrafish embryos using a pneumatic picopump (WARNER INSTRUMENTS PLI-90A).

To examine the effectiveness of MO1, we constructed a *mog1-EGFP* reporter by cloning a *mog1* cDNA fragment containing the 5′-UTR, the translation start ATG, and a part of N-terminal coding region (171 bp) in the front of *EGFP* and under the control of the *CMV* promoter. The reporter plasmid and MO1 were co-injected into embryos and the EGFP signal was captured under a florescent microscope and analyzed. To test the effectiveness of MO2, we isolated total RNA from *mog1* morphants with MO2 or Std MO, converted it into cDNA by reverse transcription, and preformed qRT-PCR analysis with the forward primer in exon 2 and the reverse primer in exon 4 as described previously^42^. The RT-PCR bands were extracted from agarose gels and sequenced by Sanger sequencing analysis to identity the nature of alternative splicing.

### Preparation and microinjection of mRNAs

The coding region of zebrafish *mog1* was PCR-amplified from cDNA prepared from embryonic mRNA, subcloned into a pGEM-T easy vector (Promega) and verified by DNA sequence analysis (pGEMT-zmog1). The zebrafish *mog1* cDNA was then subcloned into the pcs2^+^ vector, resulting in plasmid pcs2^+^-*zmog1*.

The cDNA for human *MOG1* (*hMOG1*) was amplified by PCR analysis from a mammalian expression plasmid for *MOG1* (pcDNA3.1 + MOG1)^5^,^17^. The PCR product was subcloned into the pcs2^+^ vector, resulting in plasmid pcs2^+^-*hMOG1*.

The cDNA for the coding region of the *EGFP* gene (negative control) was amplified by PCR analysis from the pEGFP-N1 vector. The PCR product was subcloned into the pcs2^+^ vector, resulting in plasmid pcs2^+^-EGFP.

The cDNA for the coding region of the human *NKX2.5* gene was amplified by PCR analysis from the pcDNA3.1−NKX2.5 expression plasmid. The PCR product was subcloned into the pcs2^+^ vector, resulting in plasmid pcs2^+^-NKX2.5.

The pcs2^+^-derived plasmids were linearized by restriction digestion with *Not I* and used for preparation of capped mRNAs for *EGFP*, *zmog1, hMOG1*, and *NKX2.5* using SP6 RNA polymerase and the mMESSAGE Mmachine system (Ambion, Austin, TX).

The mRNA samples were dissolved in water and injected into the yolk sac of one- to two-cell stage zebrafish embryos using a pneumatic picopump (WARNER INSTRUMENTS PLI-90A).

### Whole-mount *in situ* hybridization

For generation of antisense RNA probes for whole-mount *in situ* hybridization, we first used PCR to amplify a 300–600 bp cDNA fragment for a target gene to be analyzed. The cDNA fragment was subcloned into a pGEM-T easy vector. The pGEM-T easy-derived plasmids were linearized with restriction digestion. Antisense RNA probes were prepared by *in vitro* transcription from linearized templates by T7 or SP6 polymerases (Promega) in the presence of DIG-labeled nucleotides (Roche, Mannheim). Whole-mount *in situ* hybridization was performed as described by us previously^42^,^43^,^44^.

### Image acquisition

The images of embryos were visualized and captured under an Olympus SZX16 microscope with an Olympus DP72 digital camera with cellSensversion1.6 software, and processed with Adobe Photoshop CS4 soft-ware. Identical modifications and adjustments were applied to all images in the same experiment^42^,^43^,^44^.

### Quantitative real time RT-PCR analysis (qPCR)

Embryos were collected at different developmental time points (e.g. 48 hpf, 72 hpf) and used for preparation of total RNA with TRIzol reagent (TaKaRa). Genomic DNA was removed by digestion with DNA-free TMDNase (ABI, Foster City, CA). Extracted RNA samples were quantified by spectrometry. Two micrograms of RNA samples were reverse-transcribed into cDNA by reverse transcription using a cDNA Synthesis kit (Invitrogen) with random primers. The cDNA products generated using random primers were used for real time qPCR analyses of *scn5A, hcn4, kcnj2* and *cav1.3* using a FastStart Universal SYBRGreen Master kit (Roche). Real time qPCR analysis was carried out in a 10 ul reaction volume on an ABI 7900Genome Analyzer System. The *β-actin* gene was used as internal control. Data analysis was performed using the 2^−△Ct^(RQ value) method as described by us previously^45^,^46^. The sequences of primers used for real time qPCR are listed in online supplementary material (Table S1).

### Statistical analysis

An unpaired Student’s *t* test was used to compare the means from two different groups. The data were presented as means ± SEM. A *P* value of ≤0.05 was considered to be statistically significant.

## Additional Information

**How to cite this article**: Zhou, J. *et al.* Cardiac sodium channel regulator MOG1 regulates cardiac morphogenesis and rhythm. *Sci. Rep.* **6**, 21538; doi: 10.1038/srep21538 (2016).

## Supplementary Material

Supplementary Table S1

## Figures and Tables

**Table 1:** Analysis of homology between zebrafish MOG1 protein (NP_001093465) and its homologous proteins from other species.

Species	NCBI accession	Identity to zebrafish MOG1 (%)	Homology to zebrafish MOG1 (%)
Human	NP_057576	46%	62%
Mouse	NP_001272370	44%	57%
