# Supplementary material for: Cardiac sodium channel regulator MOG1 regulates cardiac morphogenesis and rhythm
Source: Sci Rep. 2016 Feb 23;6:21538. doi: 10.1038/srep21538 (PMC4763225; doi:10.1038/srep21538)
Supplement: Supplementary Table S1 [file srep21538-s1.pdf]

Cardiac sodium channel regulator MOG1 regulates cardiac morphogenesis and rhythm

Juan Zhou[1,3], Longfei Wang[1,3], Mengxia Zuo[1], Xiaojing Wang[1], Abu Shufian Ishtiaq Ahmed[1], Qiuyun Chen[2,*] and Qing K. Wang[1,2,*]

[1]Key Laboratory of Molecular Biophysics of the Ministry of Education, Cardio-X Center, College of Life Science and Technology and Center for Human Genome Research, Huazhong University of Science and Technology, Wuhan, China,

[2]Center for Cardiovascular Genetics, Department of Molecular Cardiology, Lerner Research Institute, Cleveland Clinic; Department of Molecular Medicine, Department of Genetics and Genome Sciences, Case Western Reserve University, Cleveland, Ohio, USA

[3]These authors contributed equally to this work.

*Correspondence: Qing K. Wang, Center for Human Genome Research and College of Life Science and Technology, Huazhong University of Science and Technology, 1037 Luoyu Road, Wuhan, P. R. China; Tel/Fax: +86 2787793502; Email: qkwang@mail.hust.edu.cn. or Qiuyun Chen, Center for Cardiovascular Genetics, Cleveland Clinic, Cleveland, Ohio, USA; Tel: 001 216 444 2122; Fax: 001 216 636 1231; Email: chenq3@ccf.org.

SUPPLEMENTARY MATERIAL

Table S1 PCR primers for quantitative real-time RT-PCR analysis

Gene	Primer Squence	Size
nkx2.5	Forward：5'-CTTCAGTGCTTCAGGCTTTTACGCG-3'<br>Reverse：5'-GCTCCGCATCATCCAGCTTCAGATC-3'	155 bp
gata4	Forward：5'- CGGGTGGGTTTATCCT-3'<br>Reverse：5'- ATCGCCGACTGACCTT-3'	221 bp
hand2	Forward：5'- GGACATTCTGGACAAAGATGAA-3'<br>Reverse：5'- GCCAACCAGTTCTCCCTTTA-3'	164 bp
gata5	Forward：5'- CCGGAGATGGCAACCTCATGGA-3'<br>Reverse：5'- ATTGAGCCGCAGTTCACACACTCG-3'	155 bp
cav1.3	Forward：5'- CACTCTAAAGGCTCGAAGGG-3'<br>Reverse：5'- GGCTGGAACTTGTTGGATTT-3'	144 bp
kcnj2	Forward：5'- TCCATCGAGACGCAGACTAC-3'<br>Reverse：5'- ATGCAGCCAACTATGCTTTG-3'	104 bp
hcn4	Forward：5'- TACCAGGGCAAGATGTTTGA-3'<br>Reverse：5'- CCGATGGTTCCTTCTCGTAT-3'	212 bp
scn5a	Forward：5'-GTGGATGTCCAGGAGGAAGT-3'<br>Reverse：5'- CATACCAGCATGGAGGACAC-3'	104 bp
β-actin	Forward:5'-CTCCCCTTGTTCACAATAACCTACTAATACA CAGC-3'<br>Reverse：5'- TTCTGTCCCATGCCAACCATCACTC-3'	185 bp
